# Whole-genome gene expression profiling revealed genes and pathways potentially involved in regulating interactions of soybean with cyst nematode (*Heterodera glycines* Ichinohe)

**DOI:** 10.1186/s12864-015-1316-8

**Published:** 2015-03-04

**Authors:** Jinrong Wan, Tri Vuong, Yongqing Jiao, Trupti Joshi, Hongxin Zhang, Dong Xu, Henry T Nguyen

**Affiliations:** Division of Plant Sciences and National Center for Soybean Biotechnology, University of Missouri, Columbia, MO 65211 USA; Current address: Oil Crops Research Institute, Chinese Academy of Agricultural Sciences, Wuhan, Hubei 430062 China; Department of Computer Sciences, University of Missouri, Columbia, MO 65211 USA; Informatics Institute and Christopher S. Bond Life Sciences Center, University of Missouri, Columbia, MO 65211 USA

**Keywords:** Defense, Gene expression profile, *Heterodera glycines*, Magellan, Microarray, Resistance, PI 437654, PI 567516C, Soybean, Soybean cyst nematode (SCN)

## Abstract

**Background:**

Soybean cyst nematode (SCN, *Heterodera glycines* Ichinohe) is the most devastating pathogen of soybean. Many gene expression profiling studies have been conducted to investigate the responses of soybean to the infection by this pathogen using primarily the first-generation soybean genome array that covered approximately 37,500 soybean transcripts. However, no study has been reported yet using the second-generation Affymetrix soybean whole-genome transcript array (Soybean WT array) that represents approximately 66,000 predicted soybean transcripts.

**Results:**

In the present work, the gene expression profiles of two soybean plant introductions (PIs) PI 437654 and PI 567516C (both resistant to multiple SCN HG Types) and cultivar Magellan (susceptible to SCN) were compared in the presence or absence of the SCN inoculum at 3 and 8 days post-inoculation using the Soybean WT array. Data analysis revealed that the two resistant soybean lines showed distinctive gene expression profiles from each other and from Magellan not only in response to the SCN inoculation, but also in the absence of SCN. Overall, 1,413 genes and many pathways were revealed to be differentially regulated. Among them, 297 genes were constitutively regulated in the two resistant lines (compared with Magellan) and 1,146 genes were responsive to the SCN inoculation in the three lines, with 30 genes regulated both constitutively and by SCN. In addition to the findings similar to those in the published work, many genes involved in ethylene, protein degradation, and phenylpropanoid pathways were also revealed differentially regulated in the present study. GC-rich elements (e.g., GCATGC) were found over-represented in the promoter regions of certain groups of genes. These have not been observed before, and could be new defense-responsive regulatory elements.

**Conclusions:**

Different soybean lines showed different gene expression profiles in the presence and absence of the SCN inoculum. Both inducible and constitutive gene expression may contribute to resistance to multiple SCN HG Types in the resistant soybean PI lines. Ethylene, protein degradation, and phenylpropanoid pathways, as well as many other pathways reported previously, may play important roles in mediating the soybean-SCN interactions. The revealed genes, pathways, and promoter elements can be further explored to regulate or engineer soybean for resistance to SCN.

**Electronic supplementary material:**

The online version of this article (doi:10.1186/s12864-015-1316-8) contains supplementary material, which is available to authorized users.

## Background

Annual soybean yield losses caused by soybean cyst nematode (SCN, *Heterodera glycines* Ichinohe) in the United States alone were estimated at 1.5 billion dollars [1]. One of the effective ways to control this disease is through deployment of genetic resistance in soybean cultivars. Two of the major sources of resistance employed in breeding programs are derived from soybean lines PI 88788 and Peking (PI 548402), especially the former [2]. Recently, the genes underlying two major QTL (quantitative trait loci), *Rhg1* and *Rhg4*, responsible for resistance to SCN in the soybean lines, have been cloned [3,4]. However, due to the overuse of the resistance from these sources, SCN populations showing increasing reproduction on resistant soybean cultivars have emerged [5-7]. Thus, new sources of resistance are needed for sustainable soybean production. For this purpose, a number of studies have screened plant introductions (PIs) in the USDA Soybean Germplasm Collection for new sources of resistance to SCN. As a result, new resistant lines were identified (e.g., [8-12]). Among these identified resistant lines, PI 437654 and PI 567516C were highly resistant to multiple SCN races [Races 1 (HG Type 2.5.7), 2 (HG Type 1.2.5.7), 3 (HG Type 0), 5 (HG Type 2.5.7), and 14 (HG Type 1.3.5.6.7)] [9,11,13,14]. Different from PI 437654, PI 567516C is also highly resistant to the synthetic nematode population LY1, which was derived from a mass mating of SCN races 2 (HG Type 1.2.5.7) and 3 (HG Type 0) [15,16]. This uniqueness is in agreement with the findings that PI 567516C is genetically different from most other SCN resistant lines (including PI 88788 and Peking) [17] and contains two novel QTL, which were genetically mapped on chromosomes (Chr.) 10 and 18 for resistance to multiple SCN races [14]. Although PI 437654 was shown to contain two known QTL (*Rhg1* and *Rhg4*), a new QTL for resistance to SCN HG types 0, 2.5.7, and 1.3.5.6.7 (PA 3, 5, and 14, respectively), was mapped to Chr. 20 [18]. Therefore, PI 437654 and PI 567516C are two important sources with resistance to multiple SCN HG Types, different from most other SCN resistant lines, including PI 88788 and Peking.

Microarrays are an important tool to study global gene expression in organisms, including soybean, in their growth/development processes and their responses to internal and external stimuli [19-22]. The first-generation Affymetrix Soybean Genome array covering approximately 37,500 soybean transcripts (http://media.affymetrix.com/support/technical/datasheets/soybean_datasheet.pdf) has been extensively used to probe the soybean-nematode interactions, and tremendous information has thus been generated (e.g., [23-36]). With the completion of the soybean genome sequencing project [37], the second-generation Affymetrix soybean genome array, called the Affymetrix soybean whole-genome transcript array (Soybean WT array), covering approximately 66,000 predicted soybean transcripts, was designed [38]. This array has been used to study how soybean responded to fungal infection and abiotic drought stress [38,39]. However, so far no study has been reported using this new soybean WT genome array to investigate the interactions of soybean with SCN or other nematode species. Expectedly, the application of this new array should provide a better and broader picture of soybean gene expression profiles in response to SCN due to its broader coverage of the soybean genome.

In the present work, the Soybean WT array was used to probe the gene expression profiles in the two important soybean PIs (PI 437654 and PI 567516C), which are resistant to multiple SCN HG Types, and a susceptible soybean cultivar, Magellan, in response to the SCN inoculation at two time points [3 and 8 days post-inoculation (dpi)]. These two time points roughly correspond with the initiation and establishment of syncytia, respectively [27]. Our current work revealed significant gene expression differences between these two PIs and the susceptible cultivar in response to the SCN inoculation, although some overlap was observed. Additionally, 297 genes were found constitutively expressed or suppressed in the two resistant PIs compared with the susceptible cultivar, suggesting that both constitutive and inducible gene expression may contribute to resistance to multiple SCN HG Types in the two resistant lines. Our present study revealed not only data similar to those in the published microarray work, but also some new information, such as the potential role of ethylene, protein degradation, and phenylpropanoid pathways in mediating the soybean-SCN interactions. The findings from the current study will benefit our understanding of the molecular mechanisms underlying resistance to SCN in soybean and may provide information for manipulating soybean resistance to SCN through genetic engineering.

## Results and discussion

### Gene expression profiling of the three soybean genotypes

To compare the gene expression profiles among the three soybean genotypes (PI 437654, PI 567516C, and Magellan) in response to SCN infection, 3-day-old seedlings (at the VC-Cotyledon stage) were inoculated with 2,000 J2 (juvenile 2) SCN of HG type 0 (race PA 3) [10] for 3 and 8 days, respectively. Seedlings treated with water (mock inoculation) were also collected at days 0, 3, and 8, to serve as the controls for the SCN-inoculated samples, and meanwhile to monitor constitutive gene expression in the resistant genotypes, PI 437654 and PI 567516C, in the absence of SCN by comparing with the susceptible genotype, cv. Magellan. In the present study, the SCN-regulated genes in a particular soybean line were obtained by comparing the SCN-inoculated sample with the corresponding water-treated sample at the same time point, and the constitutively-regulated genes in a particular resistant soybean line were obtained by comparing the water-treated resistant soybean line with the water-treated susceptible line (Magellan) at day 0.

Analysis of the present microarray data using dCHIP (DNA CHIP Analyzer software) [40] revealed that 3,582 genes (with a fold change ≥2 and t-test *p* value <0.05) were differentially regulated either constitutively or due to the SCN inoculation (see Additional file 1). Out of these genes, 2,375 were constitutively regulated in the two resistant PI lines by comparing with the cv. Magellan, and 1,398 genes were responsive to the SCN inoculation in the three genotypes. Interestingly, 191 genes that were regulated constitutively in the two resistant PIs were also responsive to the SCN inoculation. However, by comparing with the most recent soybean genome assembly and annotations (Glycine max Wm82.a2.v1: http://genome.jgi.doe.gov/pages/dynamicOrganismDownload.jsf?organism=PhytozomeV10), only 1,413 genes out of the 3,582 remained that were regulated either constitutively or by the SCN inoculation (see Additional file 2). Among them, 297 genes were constitutively regulated in the two resistant PI lines by comparing with cv. Magellan (see Additional file 3), 1,146 genes were responsive to the SCN inoculation in the three genotypes (see Additional file 4), and 30 that were constitutively regulated in the two resistant PIs were also responsive to the SCN inoculation (see Additional file 5). These data suggest that both constitutive and inducible gene expression may contribute to the observed resistance in the two resistant soybean PIs.

To validate the quality of the microarray data, 15 genes were randomly selected and examined for their expression using quantitative reverse transcription-polymerase chain reaction (qRT-PCR). In most cases, the qPCR results were similar to those of the microarray experiment (see Additional file 6), indicating the high quality of our microarray data.

**Figure 1:**
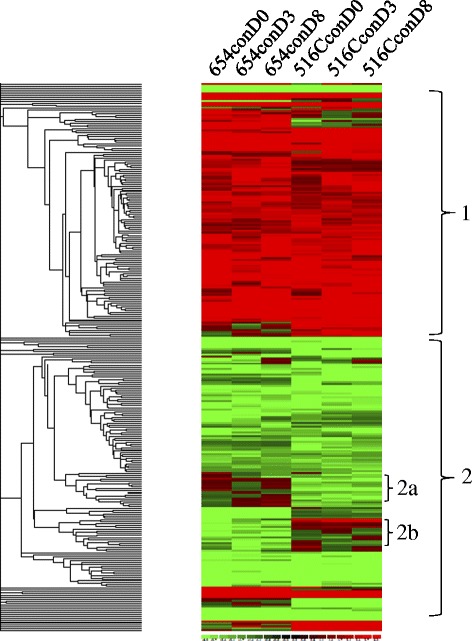
**Hierarchical clustering of the constitutively-regulated genes.** Each column represents a treatment and each row represents a gene. In total, 297 genes were constitutively regulated for at least 2 fold with a *p* value < 0.05. 654: PI 437654; 516C: PI 567516C; Mag: Magellan; con: control (mock) treatment; D0: 0 day post-inoculation; D3: 3 days post-inoculation; D8: 8 days post-inoculation. Red color: up-regulation; green color: down-regulation. The numbers on the left highlight the major clades.

**Figure 2:**
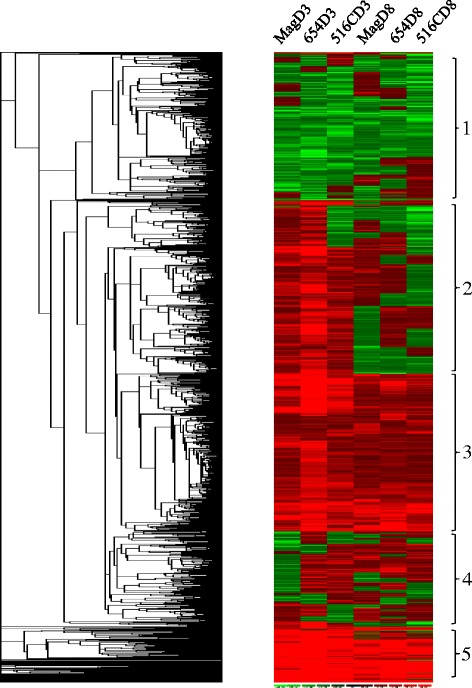
**Hierarchical clustering of the SCN-regulated genes.** Each column represents a treatment and each row represents a gene. In total, 1,146 genes were regulated by SCN for at least 2 fold with a *p* value < 0.05. 654: PI 437654; 516C: PI 567516C; Mag: Magellan; D0: 0 day post-inoculation; D3: 3 days post-inoculation; D8: 8 days post-inoculation. Red color: up-regulation; green color: down-regulation. The numbers on the left highlight the major clades.

To identify groups of co-expressed genes to reveal biological pathways and postulate transcriptional regulatory mechanisms, the constitutively regulated 297 genes were clustered using dCHIP [40]. These genes were roughly clustered into two major clades (Figure 1). Clade 1: Genes constitutively up-regulated in both PI lines, and Clade 2: Genes constitutively down-regulated in both PI lines. There are also minor clades showing unique regulations of genes in these two PI lines, e.g., clade 2a (up-regulated in PI 437654 and down-regulated in PI 567516C), and clade 2b (down-regulated in PI 437654 and up-regulated in PI 567516C). The hierarchical clustering of the SCN-regulated 1,146 genes using dCHIP [40] roughly separated them into five major clades (Figure 2). Clade 1: genes mainly down-regulated by SCN in different lines at 3 or 8 dpi; clade 2: genes mainly up-regulated in one line (primarily PI 437654) at 3 dpi; clade 3: genes up-regulated in multiple lines at 3 and/or 8 dpi; clade 4: genes with opposite expression in different lines at 3 and/or 8 dpi, and clade 5: genes up-regulated in all lines at 3 and 8 dpi.

In the following sections, we will further analyze both constitutively- and SCN-regulated genes to reveal commonality and uniqueness of gene expression between different soybean lines and pathways potentially important in regulating soybean resistance to SCN.

### Genes constitutively regulated in the two resistant PI lines

**Figure 3:**
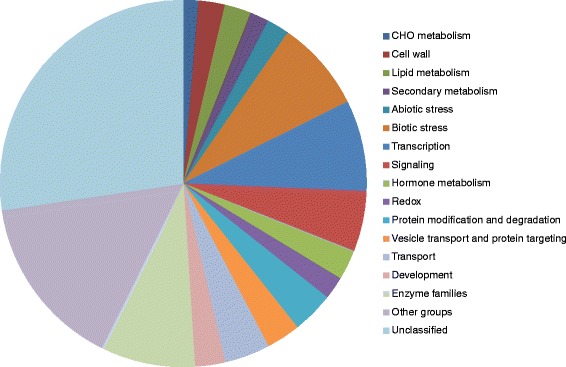
**Functional categories of the constitutively-regulated genes.** The 297 genes constitutively regulated (at least 2 fold, *p* < 0.05) in the two resistant lines were categorized using MapMan.

To reveal potentially important pathways or groups of genes involved in resistance to SCN, the constitutively-regulated 297 genes were grouped into the following functional categories using MapMan [41]: cell wall metabolism (seven genes), lipid metabolism (seven genes), secondary metabolism (five genes), abiotic stress (six genes), biotic stress (24 genes), transcription regulation (transcription factors, TFs, 24 genes), signaling (16 genes), hormonal pathways (eight genes), protein modification and degradation (11 genes), transport (12 genes), development (8 genes), enzyme families (25 genes: 4 UDP glucosyl and glucoronyl transferase genes, 3 cytochrome P450 genes, etc.), other groups of genes (46 genes: 6 genes in RNA processing, 3 genes in DNA synthesis, and 3 genes in cell organization ,etc.), and unclassified genes (82 genes) (Figure 3; see Additional file 7). It is worth noting that 23 nucleotide-binding site-leucine-rich repeat (NBS-LRR) resistance genes and one leucine-rich repeat receptor-like kinase (in the biotic stress category), seven protease and ubiquitin ligase genes (in the protein modification and degradation category), 24 TF genes (in the transcription regulation category), and four jasmonate pathway genes (in the hormone category) were constitutively regulated in the resistant PI lines, suggesting a possible role in regulating resistance to multiple SCN races in these soybean lines.

**Figure 4:**
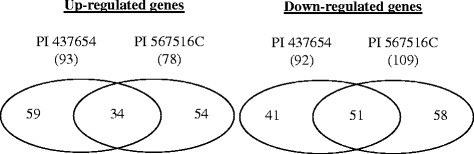
**Venn diagrams to show commonality and uniqueness of the constitutively-regulated genes between the two resistant lines.** In total, 297 genes were constitutively regulated in the two resistant soybean lines (at least 2 fold, *p* < 0.05).

Genes constitutively regulated (commonly) in both resistant PI lines: Among the 297 genes constitutively regulated in the two resistant PI lines, 34 genes were up-regulated and 51 genes down-regulated commonly in both resistant PI lines (Figure 4; see Additional file 3). For example, Glyma.01G046900 (Glyma01g05710) and Glyma.03G034400 (Glyma03g04030), both encoding an NBS-LRR type resistance protein, were highly up-regulated in both resistant lines, and genes Glyma.08G318000 (Glyma08g43020) and Glyma.08G317400 (Glyma08g42930), both also encoding an NBS-LRR type resistance protein, were significantly down-regulated in both resistant lines (see Additional file 3). These commonly regulated defense genes may play a common role, possibly in defense against SCN, in both resistant PI lines.

Genes constitutively regulated (uniquely) in PI 437654 or PI 567516C: In addition to those genes commonly regulated in both resistant PIs, there were also genes regulated differently in the two resistant PI lines: 59 genes were up-regulated and 41 genes down-regulated only in PI 437654 (Figure 4; see Additional file 3); meanwhile, 54 genes were up-regulated and 58 genes down-regulated only in PI 567516C (Figure 4; see Additional file 3). For example, Glyma.12G198100 (Glyma12g32450), encoding a protein tyrosine kinase, was significantly up-regulated in PI 437654, but not in PI 567516C; Glyma.01G115100 (Glyma01G28550), encoding a clathrin light chain, was significantly down-regulated in PI 437654, but not in PI 567516C; Glyma.13G269100 (Glyma13g34420), encoding a pathogenesis-related (PR) family protein, was significantly up-regulated in PI 567516C, but not in PI 437654; and Glyma.11G236100 (Glyma11g35990), encoding a Gamma-glutamyltranspeptidase, was significantly down-regulated in PI 567516C, but not in PI 437654. These uniquely regulated genes possibly contribute to the unique traits in these PI lines, and therefore deserve to be further studied.

### Genes regulated by the SCN inoculation in the three soybean genotypes

**Figure 5:**
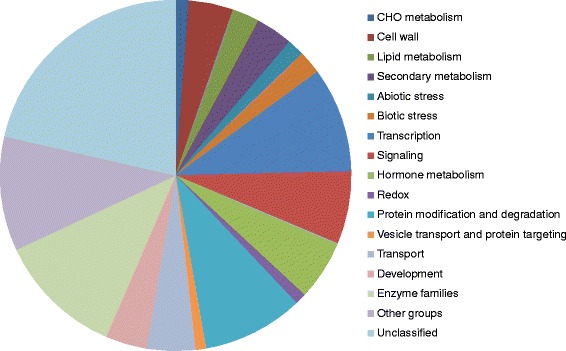
**Functional categories of the SCN-regulated genes.** The 1,146 genes regulated by SCN (at least 2 fold, *p* < 0.05) in different soybean lines were categorized using MapMan.

In addition to the constitutively-regulated genes, 1,146 genes were also regulated by the SCN inoculation in the three soybean genotypes (see Additional file 4). Once again, to get a better picture of what major gene categories and pathways were differentially regulated in the three soybean genotypes by SCN, the 1,146 genes were grouped into the following functional categories using MapMan [42]: cell wall metabolism (47 genes), secondary metabolism (39 genes), abiotic stress (18 genes), biotic stress (25 genes), transcription (111 genes), signaling (77 genes), hormone metabolism (64 genes), protein modification and degradation (107), and transport (51 genes), development (44 genes), enzyme families (133 genes: 27 cytochrome P450 genes and 25 peroxidase genes; 14 UDP glucosyl and glucoronyl transferase genes; 8 glutathione S transferase genes, etc.), other groups of genes (121 genes: 20 genes in cell organization; 10 genes in DNA synthesis; 8 genes in RNA processing and synthesis; 7 genes in cell division and cell cycle, etc.), and unclassified genes (246 genes) (Figure 5; see Additional file 8). Overall, the data suggest that many genes and pathways were affected in soybeans upon the infection by SCN. Noteworthily, genes involved in the following pathways or processes were over-represented: secondary metabolism, transcription, hormone metabolism, and protein modification and degradation. These genes and the pathways mediated by them possibly play an important role in regulating the interactions of soybean with SCN. We will discuss more about these genes and pathways later in this paper.

**Figure 6:**
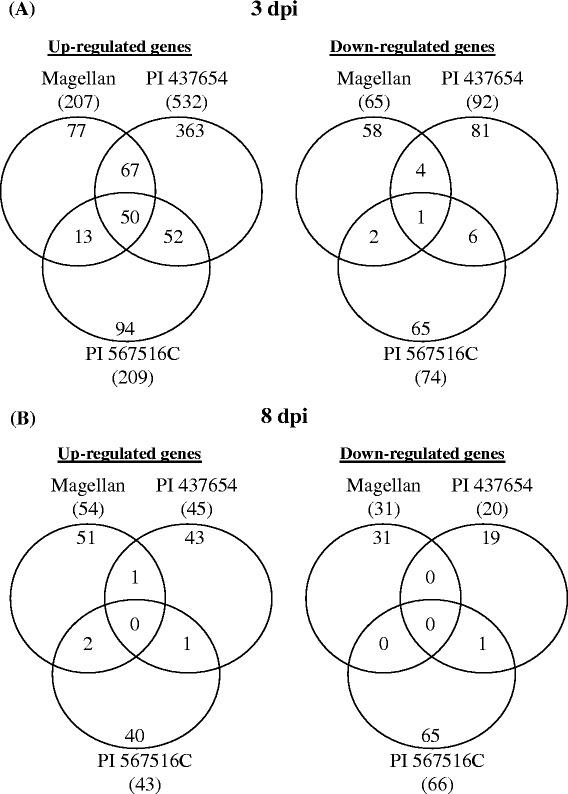
**Venn diagrams to show commonality and uniqueness of the SCN-regulated genes between different lines.** In total, 1,146 genes were regulated by SCN in different soybean lines (at least 2 fold, *p* < 0.05). **A** at 3 dpi; **B** at 8 dpi. dpi: days post-inoculation.

Genes commonly regulated by the SCN inoculation in two or three genotypes (at 3 dpi): Among the 1,146 SCN-regulated genes, 926 genes were regulated by SCN at 3 dpi (Figure 6; see Additional file 4). Among them, 50 genes were commonly up-regulated and only one gene commonly down-regulated in all three lines by the SCN inoculation at 3 dpi, e.g., Glyma.02G054200 (Glyma02g06070, encoding an S-adenosylmethionine-dependent carboxyl methyltransferase) and Glyma.05G121300 (Glyma05g24980, encoding an integral membrane protein DUF125) (Figure 6; see Additional file 4); 67 genes were commonly up-regulated and four genes commonly down-regulated only in cv. Magellan and PI 437654, e.g., Glyma.14G157700 (Glyma14g25340, encoding a protein tyrosine kinase) and Glyma.09G062600 (Glyma09g07070, encoding a Xyloglucan endo-transglycosylase C-terminus) (Figure 6; see Additional file 4); 52 genes were commonly up-regulated and six commonly down-regulated only in PI 437654 and PI 567516C, e.g., Glyma.13G208000 (Glyma13g27820, encoding an aspartyl protease) and Glyma.20G036300 (Glyma20g04840, encoding a transmembrane amino acid transporter protein) (Figure 6; see Additional file 4); and 13 genes were commonly up-regulated and 2 genes commonly down-regulated only in Magellan and PI 567516C, e.g., Glyma.18G254000 (Glyma18g48900, encoding a protein tyrosine kinase) and Glyma.03G202100 (Glyma03g36050, encoding a glycosyl transferase) (Figure 6; see Additional file 4). These commonly regulated genes may be involved in regulating general defense against SCN infection in multiple soybean lines.

Genes uniquely regulated by the SCN inoculation in individual genotypes (at 3 dpi): In addition to the commonly regulated genes by SCN in multiple soybean lines, 77 genes were up-regulated and 58 genes down-regulated only in cv. Magellan (Figure 6; see Additional file 4); 363 genes were up-regulated and 81 genes down-regulated only in PI 437654 (Figure 6; see Additional file 4); and 94 genes were up-regulated and 65 genes down-regulated only in PI 567516C (Figure 6; see Additional file 4). For example, Glyma.06G275100 (Glyma06g42750, encoding a papain family cysteine protease) was up-regulated only in cv. Magellan, and Glyma.04G247500 (Glyma 04 g42640, encoding a F-box domain protein) was down-regulated only in Magellan; Glyma.05G148300 (Glyma05g28090, encoding a calmodulin-binding protein) was up-regulated only in PI 437654), and Glyma.08G182100 (Glyma08g19410, encoding a Cytochrome P450), was down-regulated only in PI 437654; Glyma.20G148600 (Glyma20g28680, encoding a inositol 5-phosphatase) was up-regulated, and Glyma.07G010800 (Glyma07g01300, encoding a EF hand protein) was down-regulated only in PI 567516C. These uniquely regulated genes possibly contribute to the observed difference in resistance to SCN in these soybean lines.

Genes regulated by the SCN inoculation at 8 dpi: Surprisingly, only 253 genes were significantly regulated by SCN at 8 dpi, with only 33 of them also regulated by SCN at 3 dpi (Figure 6; see Additional file 4). The data suggest that different gene regulation may have occurred at the late stages of infection/defense, and SCN may also have suppressed expression of many genes to benefit infection at 8 dpi.

### Genes both constitutively- and SCN-regulated in soybean

Thirty genes that were regulated constitutively in the two resistant PIs were also responsive to the SCN inoculation (see Additional file 5), e.g., Glyma.08G256600 (Glyma08g28750, encoding an allene oxide cyclase) and Glyma.09G053700 (Glyma09g05910, encoding an ankyrin repeat-containing protein). These genes possibly play an important role in mediating the soybean-SCN interactions, and therefore may be good targets to manipulate for enhancing soybean resistance to SCN.

### Examples of gene families and pathways potentially important in regulating soybean resistance to SCN

Defense genes: Similar to published microarray studies [23-36], our present work revealed that 47 defense genes were regulated either constitutively or by SCN (see Additional file 9 and Additional file 10). Among them are five genes [e.g., Glyma.02G042500 (Glyma02g04820) and Glyma.15G206800 (Glyma15g25060)] encoding proteins similar to Arabidopsis pathogenesis-related (PR) proteins, one gene [Glyma.06G187300 (Glyma06g19900)] encoding an protein similar to Arabidopsis EDS1 (enhanced disease susceptibility 1), one gene [Glyma.06G162300 (Glyma06g17030)] encoding a protein similar to Arabidopsis RBOHD (respiratory burst oxidase protein D), one gene [Glyma.15G209300 (Glyma15g26790)] encoding a protein similar to Arabidopsis PGIP1 (polygalacturonase inhibiting protein 1, and five genes [e.g., Glyma.08G3413000 (Glyma08g45510) and Glyma.08G341400 (Glyma08g45520)] encoding trypsin and protease inhibitor family proteins. Most importantly, 28 genes [e.g., Glyma.06G268600 (Glyma06g41880) and Glyma.06G311100 (Glyma06g46810)] encode NBS-LRR type disease resistance proteins, accounting for a significant number of the total 319 NBS-LRR resistance proteins encoded by the soybean genome [42]. All this information together supports that defense genes, especially NBS-LRR resistance genes, are important in mediating soybean resistance to SCN. The defense genes revealed in the present study are possibly good targets to regulate for soybean defense against SCN. Indeed, Matthews and colleagues recently showed that the over-expression of Glyma.05G204600 (Glyma05g38130, encoding a thaumatin-like PR protein) significantly enhanced soybean resistance to SCN [43].

Transcription factors (TFs): TFs are important regulators of gene expression and are involved in plant defense against pathogens [44,45] as well as in many other events. Overall, 82 TF genes were found regulated by the SCN inoculation in the current work (see Additional file 9). Additionally, 24 TF genes were regulated constitutively in the resistant lines (three of them were also regulated by SCN) (see Additional file 9). These TF genes were primarily from the following TF families: MYB (myeloblastosis) domain-containing proteins (19 genes), basic helix-loop-helix (bHLH, 16 genes), APETALA2-ethylene-responsive element binding proteins (AP2-EREBP, 12 genes), WRKY domain transcription factors (12 genes), C2C2 zinc finger proteins (10 genes), and C2H2 zinc finger proteins (9 genes). For example, Glyma.03G002300 (Glyma03g00460), Glyma.14G103100 (Glyma14g11960), and Glyma.16G219800 (Glyma16g34590) (all encoding a WRKY TF) were significantly induced by SCN in all three genotypes; Glyma.19G262700 (Glyma19g45200, encoding an AP2-EREBP TF) was constitutively up-regulated, and Glyma.13G321900 (Glyma13g39620, encoding a putative TF) was constitutively down-regulated in both resistant lines. Considering that many similar TFs have been shown to be involved in plant defense against different pathogens in various plants [46,47], these soybean TF genes deserve further investigation to explore their possible role in mediating soybean resistance to SCN.

Protein degradation: Although many microarray studies have been done on soybean-SCN interactions, only limited information was revealed in these studies about protein ubiquitination and degradation during such interactions (e.g., [24,32]), likely due to the limited coverage of the soybean genome by the arrays used in those studies. Surprisingly, in the present study, we found that 64 genes potentially involved in protein degradation were regulated by SCN and another seven genes were constitutively regulated in two resistant lines (see Additional file 9 and Additional file 11). For example, Glyma.07G196500 (Glyma07g31630, encoding a ubiquitin-protein ligase) was constitutively up-regulated in both resistant PI lines, and Glyma.09G023700 (Glyma09g02760, encoding a ubiquitin) was constitutively down-regulated in both resistant PI lines; Glyma.18G242900 (Glyma18g47820, encoding a serine carboxypeptidase) and Glyma.09G243700 (Glyma09g37910, encoding a subtilase) were induced in all three soybean genotypes; and Glyma.02G264200 (Glyma02g43190, encoding a U-box domain-containing family protein) and Glyma.06G174800 (Glyma06g18390, encoding a cysteine proteinase) were down-regulated, especially at 3 dpi, in all three genotypes. Protein degradation is implicated in mediating plant-pathogen interactions as well as in many other processes [48-51]. However, very little is known about their involvement in mediating plant-nematode interactions. Further investigation of the genes revealed in the present work is needed to confirm their potential roles in mediating soybean-SCN interactions, and such studies could lead to effective ways to enhance soybean resistance to SCN through modulating protein degradation during soybean-SCN interactions.

Hormones: Hormones play an important role in plant-nematode interactions, as well as in plant interactions with other pathogens [52,53]. In our present study, 70 genes potentially involved in hormonal metabolism were found to be regulated constitutively and/or by SCN (see Additional file 9). Among them, 64 genes were regulated by SCN and 8 genes were constitutively regulated in two resistant lines (with two genes also regulated by SCN). Surprisingly, among them were 32 genes potentially involved in ethylene metabolism. The data suggest that ethylene and its mediated pathway may play a more important role in regulating soybean-SCN interactions than we thought, in addition to its role in regulating plant interactions with other pathogens [54-56]. Consistent with this, Tucker et al. [57] showed that the level of the ethylene precursor, 1-aminocyclopropane-1-carboxylic acid (ACC), was higher in SCN-colonized root parts than in other parts of the root, and a set of ACC synthase genes were clearly differentially expressed in SCN-colonized root parts and non-colonized roots or root tips. Recently, Fudali et al. [58] also showed that ethylene signaling modulated attraction of root-knot nematodes to Arabidopsis roots. Therefore, ethylene may be another plant hormone important in regulating soybean-SCN interactions, in addition to jasmonic acid (JA), which was well documented in the previous work [25,31,32,34,59]. In the present study, we also revealed genes potentially involved in other hormonal pathways: jasmonic acid (JA, 14 genes), auxin (9 genes), salicylic acid (SA, 5 genes), gibberellin (4 genes), abscisic acid (ABA, 2 genes), cytokinin (2 genes), and brassinosteroids (BR, 2 genes) (see Additional file 9), similar to the findings by other researchers [25,31,32]. Overall, plant hormones, especially ethylene and JA, are possibly critical in mediating soybean-SCN interactions, and further investigation is needed to understand their exact mechanisms in this process.

The phenylpropanoid pathway: The phenylpropanoid pathway plays an important role in plant growth and development as well as in biotic and abiotic stress responses, likely through regulating the formation of lignin, flavonoids, phytoalexins, etc. [60-63]. Our current microarray data showed that 20 genes potentially involved in this pathway were regulated by the SCN inoculation (see Additional file 9 and Additional file 12). For example, Glyma.01G187700 (Glyma01g39460) and Glyma.06G286600 (Glyma06g43970) (both encoding an O-methyltransferase) were significantly induced in all three lines by SCN. Consistent with our findings, previous microarray work also revealed multiple genes in the phenylpropanoid pathway regulated by the SCN inoculation [25,30]; however, our present work revealed more genes, once again, likely due to the broader coverage of the soybean genome by the array used in our study. Additionally, an early report by Edens et al. [64] showed that transcription of the genes encoding phenylalanine ammonia lyase and 4-coumaryl CoA ligase and the activities of these enzymes increased in resistant, but not in susceptible, soybean cultivars after nematode infection. And such increase in transcription and enzymatic activities led to increased synthesis of glyceollin, a phytoallaxin that inhibits multiple soybean pathogens [65]. Therefore, the phenylpropanoid pathway possibly plays a critical role in mediating soybean resistance to SCN, and further investigation is needed to understand how this pathway is involved in the process.

### Other noticeable pathways or gene families

In addition, many genes in the following important gene families or processes were also found regulated constitutively and/or by the SCN inoculation in the current study: cell wall modification (52 genes), transporters (51 genes), development (50 genes), receptor-like kinases (45 genes), Cytochrome P450 (30 genes), peroxidases (25 genes), and calcium signaling (20 genes) (see Additional file 9). These groups of genes were also revealed in many previous microarray studies (e.g., [24-36]), although generally with a smaller number, likely due to the limited coverage of the genome by the microarrays used in those studies. Further investigation of these genes will benefit our understanding of soybean-SCN interactions and may lead to effective ways to control SCN diseases in soybean.

### Promoter element analysis

To understand possible transcriptional regulation of genes revealed in the present study, promoter element analyses were conducted on the promoter regions (1,000 base pairs upstream of the start codon) of different groups of genes using the MotifSampler tool [66] incorporated into SoyKB [67,68]. All hexamer sequences in their promoter regions were examined. The most frequently occurring hexamers in most groups of genes were nTATAn and AAAAAA (data not shown). These elements likely play a general role in regulating gene expression, such as the TATA box motif with the core sequence TATA, and therefore were not of interest to us. However, a number of hexamers were significantly enriched in the promoter regions of certain groups of genes (see Additional file 13). For example, the GCATGC motif was significantly enriched in the promoters of the 58 genes similarly up- or down-regulated by SCN only in PI 437654 and PI 567516C at 3 dpi; the GyGGyG motif was significantly enriched in the promoters of the 62 genes up- or down-regulated by SCN only in PI 437654 at 8 dpi; and the rAGAGA motif was significantly enriched in the promoters of the105 genes up- or down-regulated by SCN only in PI 567516C at 8 dpi, etc. (see Additional file 10). Interestingly, these motifs all contain multiple Gs and/or Cs and appear to be novel. They are possibly involved in specific regulation of the genes revealed in the present study.

### Expression pattern of *Rhg1* and *Rhg4* in the three soybean genotypes

*Rhg1* and *Rgh4* are the two major QTL involved in soybean resistance to SCN [3,4]. Our current data showed that two out of three genes at the Rhg1 locus [3], Glyma.18G022400 (Glyma18g02580, encoding a predicted amino acid transporter) and Glyma18g02610 [encoding a protein with a WI12 (wound-inducible protein 12) region; not present in the newest soybean genome annotation], had a higher expression level in both resistant PI lines than in the susceptible cv. Magellan (2.4 ~ 4.5-fold higher) (see Additional file 14). However, the third gene, Glyma.18G022500 (Glyma18g02590, encoding an α-SNAP (α-soluble N-ethylmaleimide-sensitive factor attachment protein) vesicle-trafficking protein), was not significantly more expressed in the resistant lines, suggesting that the *Rhg1* loci in these two resistant PIs may be different from the *Rhg1* locus present in PI 88788, which shows significantly elevated expression of all three genes due to 10 copies of the 31-kb *Rhg1* repeat [3]. Consistent with this, the recent study by Cook et al. [69] showed that there were only 3 copies of the *Rhg1* repeat at the *Rhg1* locus in PI 437654 and the genes encoded by the *Rhg1* locus were moderately expressed relative to PI 88788. Therefore, our current data, together with findings by other researchers may help explain why *Rhg1* loci from soybean PI 437654 and PI 88788 respond differentially to SCN isolates [70]. *Rhg4* gene, Glyma.08G108900 (Glyma08g11490), encodes a serine hydroxymethyltransferase [4]. We found that this gene was constitutively expressed in the resistant line PI 437654 relative to the susceptible cv. Magellan (3.2 ~ 4.3-fold higher), but not in the resistant line PI 567516C. This result is in agreement with the mapping data that showed the presence of the *Rhg4* QTL in PI 437654 [18] and the absence of the typical *Rhg4* QTL in PI 567516C [14]. Further studies, such as re-sequencing the corresponding *Rhg1* and *Rhg4* regions in these PI lines, are needed to elucidate the observed differences. Some efforts are already under way in our laboratory and other laboratories (e.g., [69]).

### The present microarray work *vs*. reported microarray studies

Although so far many microarray studies have been done by different groups on the interactions of SCN with different soybean genotypes [24-36], it is not easy to compare our data directly with these previous results, due to the following reasons: different experimental conditions (different laboratories, different growth conditions, number of J2 used, and different time points, different growth stages, and different tissues or cell types), different soybean genotypes and SCN races, incompatible or compatible interactions, and different microarray platforms. However, there were a lot of similarities between our data and the published work [24-36], for example, regulation of many defense-related genes, many hormonal pathway genes, and cell wall modification genes. But results from our study appear to provide a broader picture of the gene expression profiles, either constitutively or in response to the SCN inoculation, in both resistant and susceptible soybean genotypes, due to the fact that the microarray used in our work covered many more soybean genes than those used in the previous reports. For example, we revealed more genes in ethylene pathway, protein degradation and transport processes, which could be potentially involved in mediating soybean-SCN interactions.

## Conclusions

In the present work, we compared gene expression profiles in two soybean plant introductions, PI 437654 and PI 567516C, which are resistant to multiple SCN HG Types, and one susceptible soybean cultivar, Magellan, in the presence or absence of the SCN infection. The soybean whole-genome array, which covered ~66,000 predicted soybean transcripts, was utilized for this purpose. To our knowledge, there has been no reported study on the soybean-SCN interactions using this whole genome array yet. Therefore, our current work provides a broader view of gene expression profiles in different soybean lines in the presence or absence of SCN. Overall, 297 genes were found constitutively regulated in the two resistant PI lines and 1,146 genes were found responsive to the SCN inoculation in the three soybean genotypes, with 30 genes regulated both constitutively and by SCN. These data suggest that both constitutive and inducible gene expression may contribute to the observed resistance in the two resistant soybean PI lines. Different soybean lines also showed significantly different gene expression profiles with or without the SCN inoculation. In addition to the findings similar to those reported in the published work, e.g., the regulation of many defense-related and hormonal pathway genes, we found that ethylene, protein degradation, and phenylpropanoid pathways may play an important role in mediating the soybean-SCN interactions. Additionally, we revealed multiple GC-rich motifs that may be involved in regulating gene expression in response to the SCN inoculation. Further detailed studies on selected genes and pathways may help us understand the molecular mechanisms underlying soybean resistance to SCN and may lead to effective ways to control the SCN disease in soybean.

## Methods

### Plant materials, experimental design, and inoculation

One SCN-susceptible soybean cultivar, Magellan [71], and two SCN-resistant soybean plant introductions, PI 437654 and PI 567516C, were utilized in the present study. Seeds of these soybean genotypes were germinated in germination paper pouches in the dark for 3–4 days. Each normal seedling was then transplanted into a micropot filled with steam-pasteurized sand. Seedling-containing micropots were pre-arranged in buckets, which were placed in a water bath tank with temperature maintained at 27 ± 1°C as previously described [14]. Three independent experiments were conducted using a randomized complete block design (RCBD). Ten seedlings per line were included for each replication.

A homogenous nematode population of HG type 0 (PA 3) [10] has been maintained at the University of Missouri for more than 30 generations. Eggs from females of this SCN population were incubated in 1% water agar and shaken at 25 rpm at 27°C on a rotary shaker for two days to promote hatching. Juvenile nematodes (J2) were then collected in fresh distilled water and checked for the density. For inoculation, two days after transplantation, each seedling was inoculated with 1 mL of J2 inoculum at a density of 2,000 J2/mL. In parallel, mock-inoculation with distilled water was also performed for each line as the control. Seedlings were watered daily to maintain soil moisture to facilitate uniform infection throughout the root system. Both SCN-inoculated and mock-inoculated root samples were harvested at 0, 3, and 8 days post inoculation (dpi), frozen in liquid nitrogen, and then stored at −80°C until RNA isolation. Some SCN-inoculated roots were stained with acid fuchsin [72] to confirm the successful infection.

### RNA extraction, labeling, and array hybridization

Frozen root tissue was ground to a fine powder using mortar and pestle pre-chilled in liquid nitrogen. Total RNA isolation was performed using Trizol Reagent (Invitrogen, Carlsbad, CA) following the manufacturer’s instructions. Subsequently, contaminating genomic DNA (gDNA) was removed from each sample using TURBO™ DNase following the manufacturer’s instructions (Life Technologies**,** Grand Island, NY). Approximately 500 ng of gDNA-free RNA were used to produce fragmented and biotin-labeled cDNA using the Ambion WT Expression Kit (Affymetrix, Santa Clara, CA) and the Affymetrix WT Terminal Labeling and Hybridization Kit (Affymetrix, Santa Clara, CA) following the manufacturer’s instructions. The integrity of total RNA and fragmented biotin-labeled cRNA were examined with RNA6000 Nano Assay using the Agilent 2100 Bioanalyzer™ (Agilent Technologies, Palo Alto, CA).

The microarray used in the present study was the Affymetrix Soybean Whole-Genome Transcript Array described in detail by Valdés-López et al. [38]. Hybridizations were conducted at the DNA Core Facility, University of Missouri (http://biotech.rnet.missouri.edu/dnacore), following the standard Affymetrix procedures (Affymetrix, Santa Clara, CA). The arrays were scanned with a GeneChip 7G Plus high-resolution scanner. The gene expression values were obtained using Expressionist Refiner 6.1 (GeneData) as described by Valdés-López et al. [38]. The microarray data sets used in the present study were deposited in the Gene Expression Omnibus under the accession number of GSE64492 (http://www.ncbi.nlm.nih.gov/geo/query/acc.cgi?acc=GSE64492).

### Microarray experiment and data analysis

Gene expression differences between samples were further analyzed using the software DNA-Chip Analyzer (dChip) (version release: Jan 26, 2010) [40]. The default settings were employed for normalization using the default array with median signal intensity (i.e., Mag-Ino-D8) as the baseline. Model-based expression values were computed using the default settings. To identify differentially regulated genes between two samples, the following criteria were selected: 2-fold, with a *t*-test p value <0.05, and the absolute signal intensity difference between the two samples >10. The functional classification of differential regulated genes was analyzed using MapMan [41].

### qRT-PCR analysis

cDNA synthesis and qRT-PCR reactions were performed as described [73]. The relative fold change of a gene caused by a particular treatment was calculated by first normalizing its expression to that of the reference gene (actin) in the same treated sample to obtain its normalized expression, and then comparing this normalized expression with the similarly normalized expression of the same gene in the corresponding control sample as described [74]. To calculate constitutive gene expression in the two resistant lines, the resistant lines were considered as the treated samples and the susceptible line (Magellan) as the control. The relative gene expression (fold change) was then calculated similarly.

### Promoter element analysis

The MotifSampler tool [66] incorporated into SoyKB [67,68] was used for predicting promoter consensus sequences in the 1,000 bp sequences upstream of the start codons in each group of genes. The corresponding regions of the remaining soybean genes were used as the background to infer the possible enrichment of a consensus sequence in the promoter regions of a particular group of genes. Top 10 motifs of six bases were predicted for each group of genes and were ranked based on the consensus score (cs), information content (ic) and log-likelihood (ll) [66].

## Additional files

Additional file 1:
**Master list of the 3,582 differentially regulated genes.** The table contains all the genes that were differentially regulated (at least 2 fold with a *p* value < 0.05) either constitutively or by SCN based on the old genome annotation [Glycine max genome assembly version Glyma.Wm82.a1 (Gmax1.01)]. 654: PI 437654; 516C: PI 567516C; Mag: Magellan; con: control (mock) treatment; D0: 0 day post-inoculation; D3: 3 days post-inoculation; D8: 8 days post-inoculation. Additional file 2:
**Master list of the 1,413 differentially regulated genes.** The table contains all the genes that were differentially regulated (at least 2 fold with a *p* value < 0.05) either constitutively or by SCN based on the new genome annotation [Glycine max genome assembly version Glyma.Wm82.a2.v1 (Gmax2.0)]. 654: PI 437654; 516C: PI 567516C; Mag: Magellan; con: control (mock) treatment; D0: 0 day post-inoculation; D3: 3 days post-inoculation; D8: 8 days post-inoculation. Additional file 3:
**Genes (297) constitutively regulated in two PI lines.** The table contains all the genes that were constitutively regulated (at least 2 fold with a *p* value < 0.05) in the resistant PI lines (PI 437654 and PI 567516C) by comparing with the susceptible cultivar Magellan. 654: PI 437654; 516C: PI 567516C; Mag: Magellan; con: control (mock) treatment; D0: 0 day post-inoculation; D3: 3 days post-inoculation; D8: 8 days post-inoculation. Black: Genes (85) constitutively up- or down-regulated in both PI lines (PI654 and PI516C); Red: Genes (100) constitutively up- or down-regulated only in PI654; Green: Genes (112) constitutively up- or down-regulated only in PI516C. Additional file 4:
**Genes (1,146) regulated by SCN in different lines.** The table contains all the genes that were differentially regulated (at least 2 fold with a *p* value < 0.05) by SCN in any soybean line at 3 and/or 8 days post-inoculation. 654: PI 437654; 516C: PI 567516C; Mag: Magellan; con: control (mock) treatment; D0: 0 day post-inoculation; D3: 3 days post-inoculation; D8: 8 days post-inoculation. Black: Genes (51) similarly up- or down-regulated in all three lines at 3 dpi; Dark red: Genes (71) similarly up- or down-regulated only in both Mag and PI654 at 3 dpi; Red: Genes (58) similarly up- or down-regulated only in both resistant lines at 3 dpi; Orange: Genes (15) similarly up- or down-regulated only in both Mag and PI516C at 3 dpi; Yellow: Genes (135) up- or down-regulated only in Mag at 3 dpi; Light green: Genes (444) up- or down-regulated only in PI654 at 3 dpi; Green: Genes (159) up- or down-regulated only in PI516C at 3 dpi; Light blue: Genes (5) similarly up- or down-regulated in 2 or 3 lines at 8 dpi; Blue: Genes (82) up- or down-regulated only in Mag at 8 dpi; Dark blue: Genes (62) up- or down-regulated only in PI654 at 8 dpi; Purple: Genes (105) up- or down-regulated only in PI516C at 8 dpi; Light purple: Genes (33) commonly regulated at both 3 and 8 dpi. Additional file 5:
**Genes (30) regulated both constitutively and by SCN.** The table contains all the genes that were regulated (at least 2 fold with a *p* value < 0.05) both constitutively and by SCN in the resistant soybean lines (PI 437654 and PI 567516C). 654: PI 437654; 516C: PI 567516C; Mag: Magellan; con: control (mock) treatment; D0: 0 day post-inoculation; D3: 3 days post-inoculation; D8: 8 days post-inoculation. Additional file 6:
**Comparison of microarray and qPCR data.** Fifteen genes were randomly selected from the master list of the genes differentially regulated (see Additional file 1), and examined for their expression at 3 and 8 days post-inoculation using qPCR. The results were the average from three biological replicates. Additional file 7:
**Functional categories of constitutively regulated genes.** The constitutively-regulated 297 genes were grouped into different functional categories using MapMan. Additional file 8:
**Functional categories of SCN-regulated genes.** The SCN-regulated 1146 genes were grouped into different functional categories using MapMan. Additional file 9:
**Selected groups of genes.** The table contains the selected gene families or groups that were significantly represented in the differentially regulated genes. 654: PI 437654; 516C: PI 567516C; Mag: Magellan; con: control (mock) treatment; D0: 0 day post-inoculation; D3: 3 days post-inoculation; D8: 8 days post-inoculation. 654: PI 437654; 516C: PI 567516C; Mag: Magellan; con: control (mock) treatment; D0: 0 day post-inoculation; D3: 3 days post-inoculation; D8: 8 days post-inoculation. Additional file 10:
**Biotic stress pathways.** Many genes possibly involved in multiple biotic stress pathways were differentially regulated either constitutively or by SCN. The pathways were generated using MapMan. Additional file 11:
**Ubiquitin-dependent protein degradation pathway.** Many genes possibly involved in the ubiquitin-dependent protein degradation pathway were differentially regulated either constitutively or by SCN. The pathway was generated using MapMan. Additional file 12:
**The phenylpropanoid pathway.** Many genes possibly involved in the phenylpropanoid pathway were differentially regulated either constitutively or by SCN. The pathway was generated using MapMan. Additional file 13:
**Occurrence of G-rich elements in different groups of genes.** The MotifSampler tool was used to reveal promoter elements possibly enriched in the promoter regions of certain groups of genes that were differentially regulated. The corresponding regions of the remaining soybean genes were used as the background to infer the possible enrichment of an element in the promoter regions of a particular group of genes. Additional file 14:
**The Rhg1 and Rhg4 genes.** The expression patterns of the Rhg1 and Rhg4 genes in the two resistant soybean lines. 654: PI 437654; 516C: PI 567516C; Mag: Magellan; con: control (mock) treatment; D0: 0 day post-inoculation; D3: 3 days post-inoculation; D8: 8 days post-inoculation.
