# Supplementary figures and images for: Whole-genome gene expression profiling revealed genes and pathways potentially involved in regulating interactions of soybean with cyst nematode (Heterodera glycines Ichinohe)
Source: BMC Genomics. 2015 Mar 4;16(1):148. doi: 10.1186/s12864-015-1316-8 (PMC4351908; doi:10.1186/s12864-015-1316-8)

## Slide 1
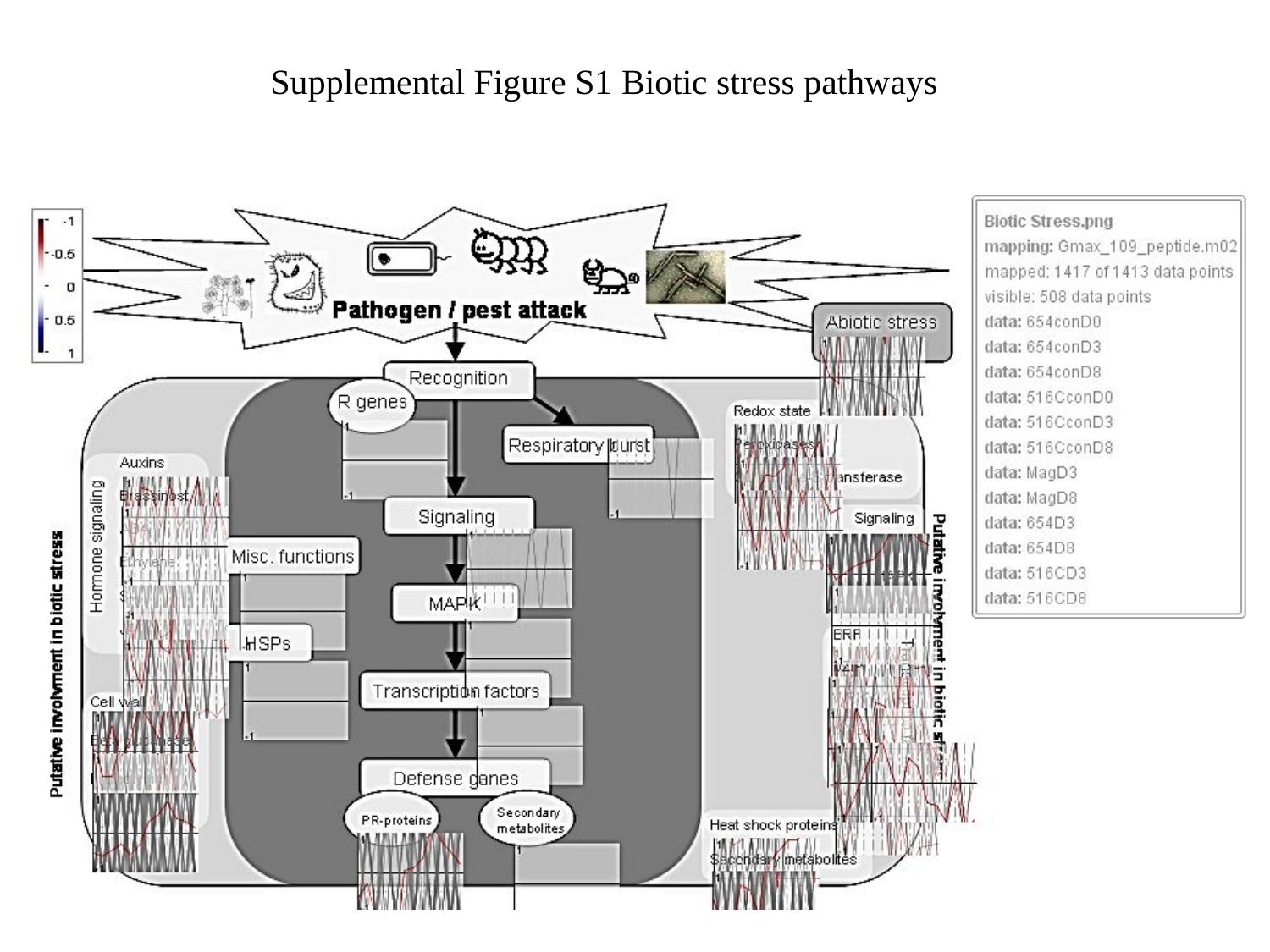

Supplemental Figure S1 Biotic stress pathways

Supplement: Additional file 10: — Biotic stress pathways. Many genes possibly involved in multiple biotic stress pathways were differentially regulated either constitutively or by SCN. The pathways were generated using MapMan. [file 12864_2015_1316_MOESM10_ESM.pptx]

## Slide 1
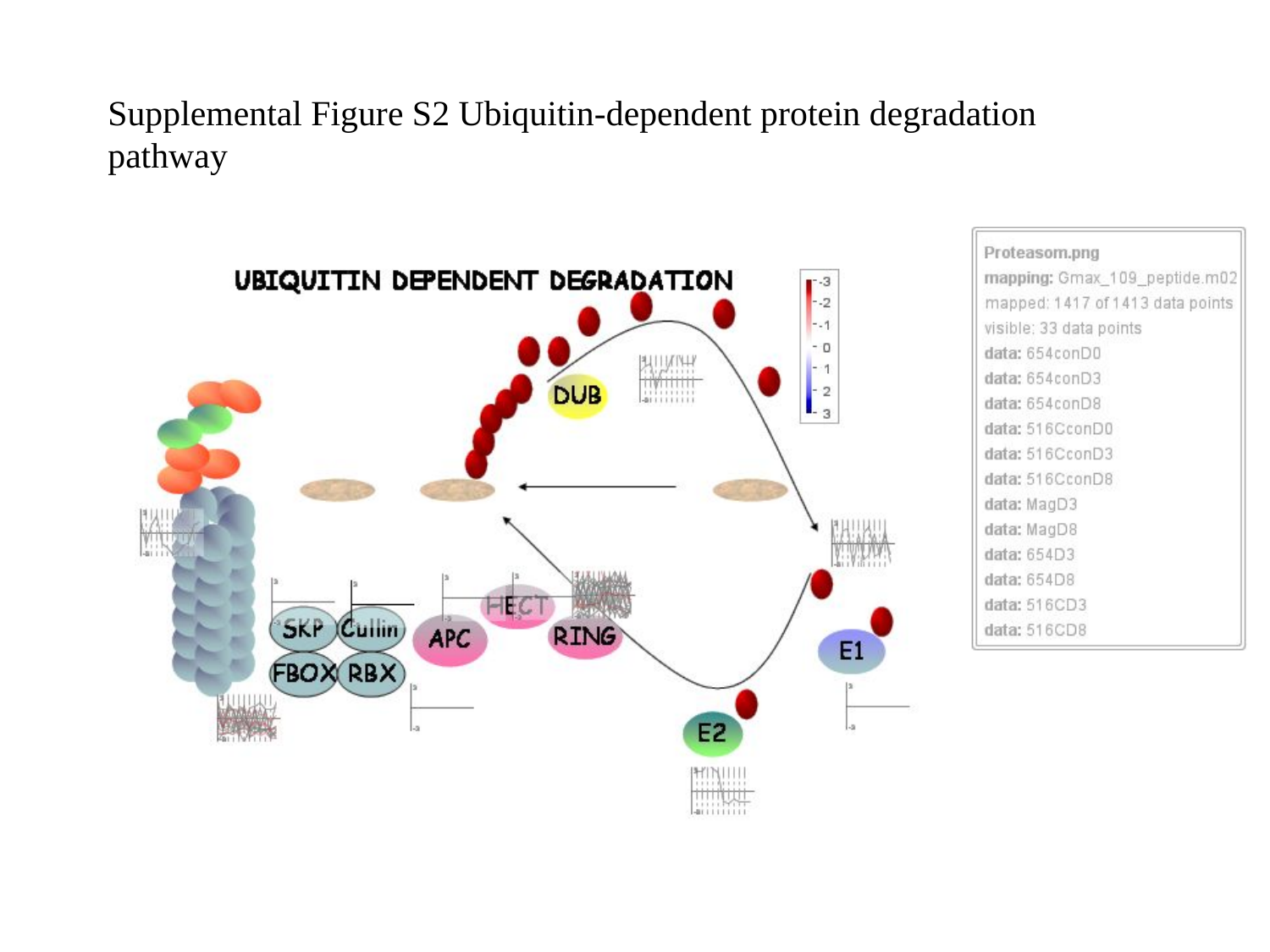

Supplemental Figure S2 Ubiquitin-dependent protein degradation pathway

Supplement: Additional file 11: — Ubiquitin-dependent protein degradation pathway. Many genes possibly involved in the ubiquitin-dependent protein degradation pathway were differentially regulated either constitutively or by SCN. The pathway was generated using MapMan. [file 12864_2015_1316_MOESM11_ESM.pptx]

## Slide 1
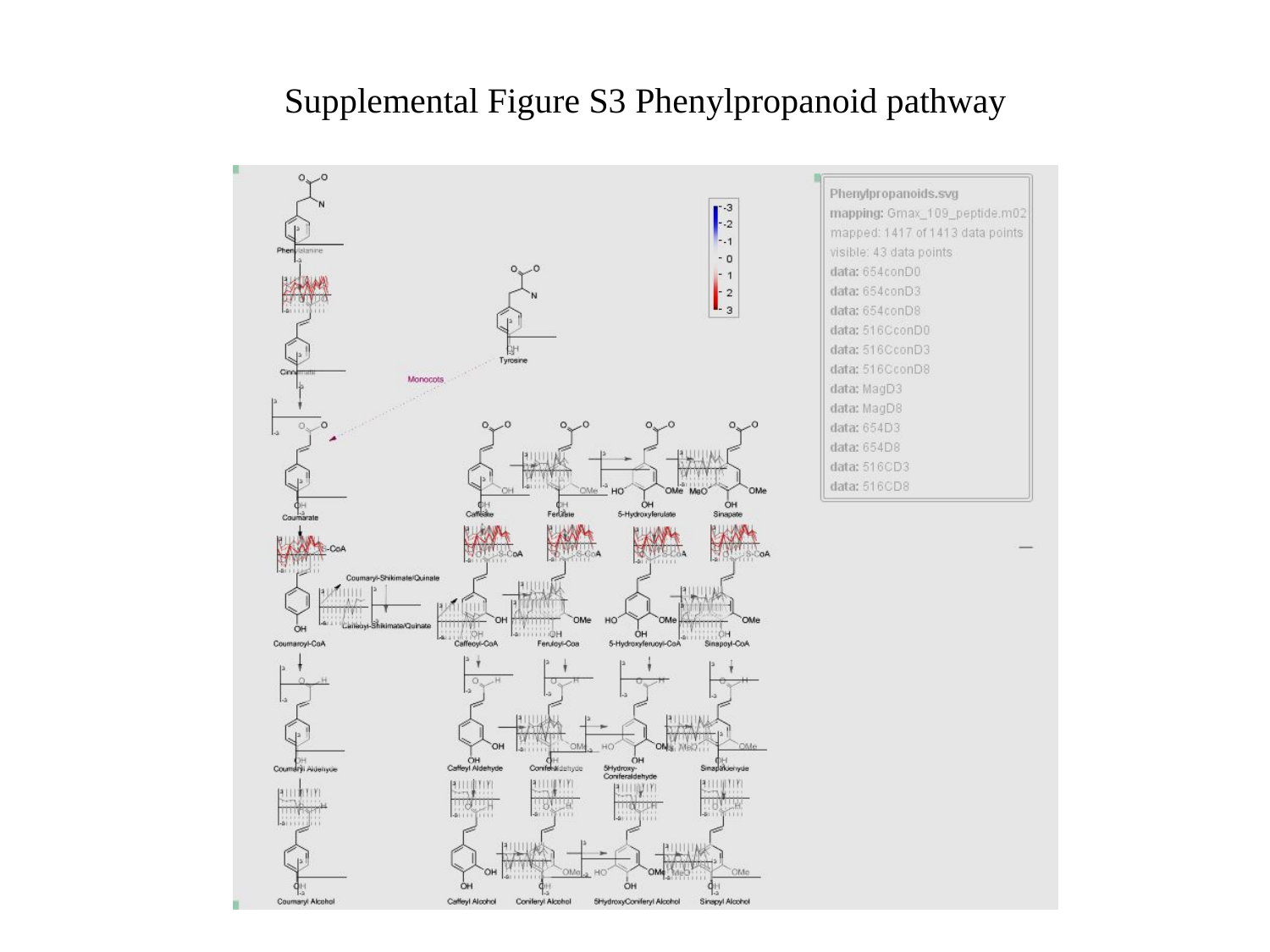

Supplemental Figure S3 Phenylpropanoid pathway

Supplement: Additional file 12: — The phenylpropanoid pathway. Many genes possibly involved in the phenylpropanoid pathway were differentially regulated either constitutively or by SCN. The pathway was generated using MapMan. [file 12864_2015_1316_MOESM12_ESM.pptx]
